# Developing transformative capacity through systematic assessments and visualization of urban climate transitions

**DOI:** 10.1007/s13280-018-1109-9

**Published:** 2018-11-03

**Authors:** Erik Glaas, Mattias Hjerpe, Sofie Storbjörk, Tina-Simone Neset, Anna Bohman, Prithiviraj Muthumanickam, Jimmy Johansson

**Affiliations:** 1 Department of Thematic Studies–Environmental Change, Centre for Climate Science and Policy Research, Linköping University, 581 83 Linköping, Sweden; 2 Department of Science and Technology, Media and Information Technology, Linköping University, 601 74 Norrköping, Sweden

**Keywords:** Assessment, Climate change, Governance, Transformative capacity, Urban Climate Transition, Visualization

## Abstract

Transforming cities into low-carbon, resilient, and sustainable places will require action encompassing most segments of society. However, local governments struggle to overview and assess all ongoing climate activities in a city, constraining well-informed decision-making and transformative capacity. This paper proposes and tests an assessment framework developed to visualize the implementation of urban climate transition (UCT). Integrating key transition activities and process progression, the framework was applied to three Swedish cities. Climate coordinators and municipal councillors evaluated the visual UCT representations. Results indicate that their understanding of UCT actions and implementation bottlenecks became clearer, making transition more governable. To facilitate UCT, involving external actors and shifting priorities between areas were found to be key. The visual UCT representations improved system awareness and memory, building local transformative capacity. The study recommends systematic assessment and visualization of process progression as a promising method to facilitate UCT governance, but potentially also broader sustainability transitions.

**Electronic supplementary material:**

The online version of this article (10.1007/s13280-018-1109-9) contains supplementary material, which is available to authorized users.

## Introduction

Cities and local governments are described as seedbeds for transformation into climate-proof, low-carbon, and sustainable societies (Viguie and Hallegatte 2012; Lee and Painter 2015). While numerous climate activities are being implemented, fundamental transformation will require integrated approaches across sectoral divisions and actor groups (Moloney and Horne 2015), and holistic ways to plan for and govern urban systems (Wolfram et al. 2017). Accordingly, it is becoming more pertinent for local governments to overview their current activities and assess if and to what extent the city is transitioning to enable governance of these processes (Wamsler et al. 2014).

This paper focuses on method development to support governance of urban climate transitions (UCT), defined as “processes in which both the technical and social parts of the system transform in order to tackle climate change” (Boyd and Juhola 2015, p. 1239). By analyzing how comprehensible overviews of UCT process progression can be created through assessing and visualizing current transformative climate action, and how resulting visual representations can influence governance, this study contributes to the understanding of how to develop urban transformative capacity. The urban transformation and transition literature suggests three reasons why developing more comprehensive and transparent ways to assess UCT progress are needed.

First, UCT are complex and highly context-specific processes (Romero-Lankao and Gnatz 2013; Burch et al. 2014). Local governments’ climate responses have largely been voluntary and, thus, taken different shapes, referred to as a patchwork (Bulkeley et al. 2012; Moloney and Horne 2015). Responses typically occur across a range of sectors: energy supply, mobility, water supply, urban planning, health- and elderly care, etc., and are managed by several actors (Hoppe and van Bueren 2015). As Romero-Lankao (2012) notes, most studies of local climate activity have adopted a sectorial approach, with few accounts covering the breadth of responses.

Second, the transition and transformation concepts have been advanced and influenced by different literature studies, highlighting different elements of the transition process (Boyd and Juhola 2015; Feola 2015; Hjerpe et al. 2017). Until recently, these concepts have evolved in relative isolation. The introduction of the Transformative Capacity concept (Wolfram 2016; Pahl-Wostl 2017; Hölscher et al. 2018) is one attempt to amass the capacities needed to transform, cutting across different conceptualizations. How to advance these capacities in local climate governance is, however, still not clear.

Third, methods to assess the progress of transformation, i.e., whether the ensemble of implemented urban climate activities is pointing towards transformation, are lacking (Turnheim et al. 2015; Feola 2015; Hjerpe et al. 2017). Even if sustainability is generally considered to be the target, the UCT process progression needs to be more systematically assessed to illuminate how current decisions contribute to achieve this target.

The above complexity, ambiguity, and lack of methods to assess progress suggest that, at present, gaining a processual understanding of UCT is challenging, and ultimately constrains well-informed, strategic decision-making. This paper evaluates whether and how systematic assessments and visualization of UCT progression can improve urban transformative capacity in local climate governance. We propose and test an assessment framework developed to visualize UCT progression across sectors and actors. Three research questions have guided the study:

1. What elements should be included to systematically assess and visualize UCT processes?
2. What patterns of local UCTs can be identified through visual representation of implemented climate actions?
3. How can visual representations of UCTs influence the transformative capacity in local governance?

Application of the assessment framework and interpretations of its results with key actors in three Swedish cities are used to discuss how such assessments and visualization of transition processes can influence transformative capacity in local climate governance.

The paper is structured as follows: the subsequent section details how the UCT assessment framework and its visualization components were constructed using literature surveys, and how their influence on local transformative capacity was evaluated; the next section outlines how the framework was applied and tested within three Swedish cities, followed by a discussion regarding what the UCT representations show, and whether and how the representations can influence the transformative capacity in local governance. Finally, the paper concludes by outlining how systematic assessments and visualization of transition processes can be used and further researched.

## Developing the assessment framework and evaluating its influence on transformative capacity

UCT processes are highly complex and include transformative mitigation and adaptation actions among various actors, sectors, and implementation logics (Viguie and Hallegatte 2012). Representing the scope and progress of an UCT process—making it easier to grasp but still not over-simplified—is challenging, yet necessary to enable comparisons across time and space (Lee and Painter 2015). We have surveyed literature on transformative climate action, sustainability transitions and transformations, and process visualization to establish the assessment and visualization framework identifying: (1) what activities are needed, referred to as *key urban climate transition activities* for which a local government has a direct or indirect mandate to steer implementation, (2) how far current activity has progressed, referred to as *process progression indicators* for deliberate UCT actions, and (3) how UCT should be represented via process visualization focused on *static representations*.

### Key UCT activities

Key UCT activities were identified by surveying urban climate mitigation and adaptation studies retrieved from the Scopus database (see Table S1), resulting in 201 articles covering a wide geographical spread and scholarly positions. Of these, articles 98 were targeting intended climate actions, as opposed to spontaneous actions or biological processes. These were analyzed in depth.

To support generic applicability, we included mitigation and adaptation activities that were found significant for UCT in at least two locations. We found 36 such activities, representing the scope of UCT, and merged these into eight thematic areas (Table 1). The full references are included in Electronic Supplementary Material.

**Table 1:** Identified key UCT activities merged into eight thematic areas (full Table S5 and references in Electronic Supplementary Material)

Area	Transition activities
Energy	1. Support energy saving among individuals and companies
	2. Optimize waste management
	3. Decrease the use of non-renewable energy
	4. Increase the share of renewable energy
	5. Develop effective district heating and cooling
	6. Adaptation of energy system, grid, and IT
Transport	7. Reduce GHG emissions from passenger transports
	8. Reduce GHG emissions from goods transports
	9. Increase the share of public transportation, biking, and walking
	10. Adaptation of roads and transport infrastructure
Building and housing	11. Support sustainable land use through urban densification
	12. Increase energy efficiency in buildings
	13. Decrease emissions from constructions
	14. Adaptation of official buildings and information to private house owners
	15. Adaptation of cultural heritage (e.g., buildings with cultural values)
Planning and governance	16. Mitigation considerations inherent in urban planning
	17. Cooperation with citizens and companies for resilience and low GHG emissions
	18. Adaptation considerations inherent in urban planning
	19. Increase share of green–blue infrastructure
	20. Holistic flood risk management
	21. Inter-municipal cooperation and learning for resilience and low GHG
	22. Adaptation of tourism in a changing climate
Agriculture and forestry	23. Decrease GHG emissions from agriculture and forestry
	24. Enhance usage of locally produced food and timber
	25. Adaptation of agriculture and forestry on own land or info. to producers
	26. Facilitate urban and peri-urban agriculture and gardening
Biodiversity	27. Increase the share of organic food (schools, health care)
	28. Mainstream ecosystem-based adaptation in environmental management
	29. Preserve biological diversity in a changing climate
Health	30. Identify vulnerable groups (for heat, flooding, etc.)
	31. Adaptation to avoid health related impacts (for heat, flooding, etc.)
	32. Adapt management practices in health and social care
Water infrastructure	33. Assess vulnerability of and adapt urban storm and waste water systems
	34. Assess vulnerability of and adapt drinking water systems
	35. Secure reserve water (in case of, e.g., drought or contamination)
	36. Decrease leakage in water infrastructure

The assessment framework incorporates these activities to elucidate specific UCT actions. It does not, however, explicitly deal with interactions between activities (Viguie and Hallegatte 2012), which nevertheless were discussed during the tests.

### Process progression

Process progression indicators are used to assess how far the implementation of climate action has come for each key activity. As transformation involves fundamental change, scholars have often approached transition as a process (Feola 2015), distinguishing between different process phases. Moore et al. (2014) suggest four phases: pre-transformation, preparing for change, navigating the transition, and institutionalizing the new trajectory. Other scholars outline more detailed UCT process phases: problem structuring, envisioning, and establishing a transition arena; developing coalitions and transition agendas; mobilizing actors and executing projects; and evaluating and learning (Loorbach 2010; Nevens and Roorda 2014).

To derive an evaluation scheme for UCT process progression, we merged the process phases from the above literature, providing complementing perspectives (Table 2). Our evaluation system distinguishes between, firstly, three main phases: *initiation*, *innovating*, and *scaling*-*up* and, secondly, the spread of action within and outside the local municipal administration (Table 2).

**Table 2:** Evaluation system for UCT process evolvement. Process progression is displayed in Figs. 1 and 2 by deeper color shades. The inner circle corresponds to the initiating phase, the middle circle to the innovating phase, and the outer circle to the scaling-up phase

Phase	Actions taken		Actors targeted			
	Process indicator		0 points	+1 point	+1 point	+1 point
Initiation	Issue raised	Acknowledging need for action	No account taken	Issue raised and/or investigated	Internal goals, plan, and/or cooperation developed	External goals, plan, and/or cooperation developed
	Investigation	Assessment of risks and actions				
	Goal	UCT vision or goal formulated				
	Plan	Planned activities/instruments				
	Cooperation	Involvement of key stakeholders				
Innovating	Guideline	Instructions for action developed	No concrete action	Internal guidelines and/or services implemented	Internal responses and/or experiments implemented	External guideline, services responses, and/or experiments implemented
	Service	Support for UCT implementation				
	Response	Well-known measures implemented				
	Experiment	New measures implemented				
Scaling-up	New procedure	New responses, guidelines or services mainstreamed and spread	No up-scaling activities	Limited internal new procedures implemented	Far-reaching internal new procedures implemented	External new procedures implemented

UCT actions corresponding to the *initiating* phase includes raising an issue and problem structuring by investigating the climate challenge from several perspectives (Moore et al. 2014; Nevens and Roorda 2014). *Initiating* further comprises stakeholder involvement (Burch et al. 2014; Moloney and Horne 2015) and policy formation (Burch et al. 2014). Studies of urban climate governance have empirically demonstrated significant variation in the degree of institutionalization in the policy developed (Burch et al. 2014; Nevens and Roorda 2014; Moloney and Horne 2015; Wolfram 2016). We thus distinguish between preparatory work, such as raising and investigating an issue, and clearly articulated goals (Burch et al. 2014) and encircling actions into a designated plan (Loorbach 2010; Turnheim et al. 2015).

UCT actions associated with the *innovating* phase concerns implementing concrete actions, including experiments and proposed transformative physical and policy responses (Bulkeley and Castan-Broto 2013; Nevens and Roorda 2014). It also involves guidelines or services put in place to support and empower UCT involvement, such as energy advice or information campaigns (Ziervogel et al. 2016).

UCT actions indicating *scaling*-*up* includes broader implementation of successful experiments or responses as new procedures in the organization, c.f. mainstreaming (Nevens and Roorda 2014), and spreading them to other actors in the city or to other cities to increase systemic coverage. Mobilization of resources has been found critical to enable *scaling*-*up* and eventually overcoming the large inertia of current systems (Moore et al. 2014; Moloney and Horne 2015; Hrelja et al. 2015).

Scores have been assigned for all key activities and each process phase individually (Table 2). We have assessed the actions taken and the actors targeted, assigning numbers from 0 to 3. A “0” is assigned when no activity was found. A “1” is assigned when activity is limited, i.e., if experiments and responses have only been implemented in one department or a small part of the system. A “2” is assigned when activity is internal, meaning that it applies to the whole municipal organization, i.e., when experiment(s) or response(s) is spread to all relevant parts of the municipal organization. A “3” is assigned when the activity applies to relevant non-municipal actors, i.e., when goals and plans target both municipal and non-municipal actors. The complete scores are displayed in Tables S2–S4.

### Process visualization

Process visualization can be applied to analyze and overview complex processes, producing easily accessible information on performance (Matković et al. 2002). As of yet, most applications of process visualization focus on industrial processes, for instance describing production chains (Al-Kassab et al. 2014), whereas visual representations of process progression within organizations are rare. *Process visualization techniques* were identified by surveying studies retrieved from the Scopus database using the search terms “process visualization” and “organization,” resulting in 26 articles analyzed in depth. Promising process visualization techniques were assessed and tested for their applicability to represent the key UCT activities and progression in the case cities using side-by-side comparison (Low et al. 2017). Examples included bar diagrams, line charts, decision trees, flowcharts, strategy maps, and tracking diagrams (Eppler and Platts 2009).

**Fig. 1:**
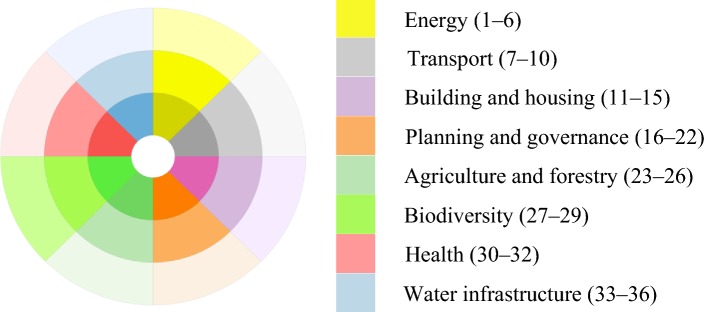
Visualization principle with the eight thematic UCT areas and the three UCT process progression steps. Each thematic area is assigned a color. The thematic area is made up of three to seven key UCT activities according to the numbering in Table 1. The three concentric circles represent the UCT process phases; the inner circle represents the initiating phase, the middle circle the innovating phase, and the outer circle the scaling-up phase. Color intensity represents the scores for each area and process step set according to the evaluation system (Table 2). Darker color shades indicate more progress. Inaction is represented by a white zone

The Florence Nightingale chart, also referred to as a rose diagram or polar area chart (Draper et al. 2009), stood out as particularly useful for visualizing UCT processes. This technique is a version of the commonly used pie chart with the main difference, however, that each zone of the Nightingale chart is equiangular (Gupta et al. 2016). Accordingly, differences in the zones are displayed by different radiuses rather than different angular magnitudes. This technique has previously been applied in sustainability research where progress in different categories is compared without emphasizing one category over the other (c.f. Rockström et al. 2009). Since comparison rather than ranking is made between key transition areas, this technique was considered suitable. Key transition areas and UCT process progression can be represented with reduced complexity by giving all areas equal weight, allowing efficient analysis of different levels of progression, and to rapidly identify parts where no progress has been made. The charts thus are set up to enable inclusive dialogues between stakeholders (Fig. 1).

### Evaluating the influence of UCT representations on transformative capacity

Contemporary transformation and transition literature emphasizes the limited capacity of governance systems to “decisively shift societal development towards low-carbon, sustainable and resilient futures” (Hölscher et al. 2018, p. 2). Outlining the capacities and governance processes needed, scholars in related research fields have compiled factors suggested in studies as significant for transforming current governance under the heading of Transformative Capacity. To evaluate whether and how the above presented UCT assessment framework can influence the transformative capacity in local climate governance, we have merged factors proposed in three recent frameworks of transformative capacity targeting urban governance (Wolfram 2016), water governance (Pahl-Wostl 2017), and climate governance (Hölscher et al. 2018). These seven broad factors are used as evaluation criteria for analyzing in what way the UCT representation can influence transformative capacity in local climate governance (Table 3).

**Table 3:** Evaluation criteria and factors of transformative capacity

Evaluation criteria	Factors	Author(s)
A. Foster new forms of governance and leadership	Diverse governance modes	Wolfram (2016)
	Combination of governance modes	Pahl-Wostl (2017)
	Strengthening self-organization	Hölscher et al. (2018)
	Balance top-down and bottom-up processes	Pahl-Wostl (2017)
	Transformative leadership	Wolfram (2016)
B. Engage and empower stakeholders	Participation and inclusiveness	Wolfram (2016)
	Sustained intermediaries	Wolfram (2016)
	Empowered and autonomous communities of practice	Wolfram (2016)
	Informal networks	Pahl-Wostl (2017)
	Mediating across scales and sectors	Hölscher et al. (2018)
C. Create shared visions	Urban sustainability foresight	Wolfram (2016)
	Strategic alignment	Hölscher et al. (2018)
	Breaking open resistance to change	Hölscher et al. (2018)
D. Develop system overview	System(s) awareness and memory	Wolfram (2016)
	Generating knowledge about system dynamics	Hölscher et al. (2018)
E. Facilitate experimenting and innovation	Diverse community-based experimentation	Wolfram (2016)
	Innovation embedding and coupling	Wolfram (2016)
	Enabling novelty creation	Hölscher et al. (2018)
	Increasing visibility of novelty	Hölscher et al. (2018)
F. Spur reflexivity and monitoring of progress	Reflexivity and social learning	Wolfram (2016)
	Monitoring and continuous learning	Hölscher et al. (2018)
	Revealing unsustainable path dependencies	Hölscher et al. (2018)
G. Scale-up and embed implementation	Working across human agency levels	Wolfram (2016)
	Working across political-administrative levels and geographical scales	Wolfram (2016)
	Creating opportunity contexts	Hölscher et al. (2018)
	Polycentric structures with flexible coordination	Pahl-Wostl (2017)

## Methods and materials

The assessment framework was applied and tested in three case cities located in Östergötland county, Sweden: Finspång, Linköping, and Norrköping. The cities differ in terms of economic and demographic structure to get a spread in results (Table 4), while sharing similar regulatory frameworks by being situated in the same county. This means that differences in the visual representations mirror only internal choices made.

**Table 4:** Characteristics of the case cities

City	Population	Location	Economic function
Finspång	20 000	Inland	Industrial
Linköping	155 000	Inland	Administration and knowledge center
Norrköping	140 000	Coastal	Logistical and knowledge center

Different sets of materials and methods were used to

- apply our assessment framework in the cities, i.e., to produce the visual UCT representations, and
- test the visual UCT representations with municipal climate coordinators and municipal councillors, i.e., stakeholders mandated to govern and coordinate urban climate and sustainability actions strategically.

### Applying the assessment framework

Secondary data and structured interviews with sector-specific staff were used to identify concrete actions within the 36 key UCT activities (Table 1). Secondary data included comprehensive plans, energy plans, nature preservation programs, departmental management plans, environment and climate policies (Tables S2–S4). Structured interviews were held with eight officials from the sectors for which the climate coordinator did not have full insights into the climate-related work conducted, or in case the secondary data lacked enough detail. Mostly these officials represented the water, planning, and environment departments or utilities, but shifted among the municipalities. For each activity and process step, scores were set according to the evaluation system (Table 2) and the visual UCT representations were produced. Strictly following a uniform assessment approach simplified the identification of actions taken. Although the approach risks missing climate action falling outside the scope of the 36 key activities, the systematic assessment facilitated comparison only of actions described as transformative.

### Evaluating the UCT representations

Following an established approach for evaluating climate-related visualization tools (Glaas et al. 2017), discussions with municipal stakeholders were arranged. Individual interviews were held with officials mandated to coordinate municipal climate action in two steps: first to validate and complement the data collections as above, and secondly to evaluate the UCT representations. Additionally, in Norrköping, a workshop was held with six municipal councillors and their political secretaries to get the political governor’s perspective. Open-ended questions targeted perceived challenges in the UCT work, current collaboration with other actors, and validity and usefulness of the visual UCT representations. The interviews and the workshop lasted approximately 1.5 h and were recorded and transcribed. We analyzed the transcripts by meanings concentration, emphasizing reoccurring featured themes, including overview, usefulness, effectiveness, significance, and target for UCT. When presenting the empirical results, statements and reflections are included to support and illustrate our findings (Silverman 1993).

## Results and discussion

Applying and testing the assessment framework provided insights into how the visual UCT representations can be interpreted, the role of system overview in local climate governance, and the usefulness and need for further development of the framework as below. At the end of this section, we discuss how the UCT representations can influence local transformative capacity.

### Local applications of the assessment framework

**Fig. 2:**
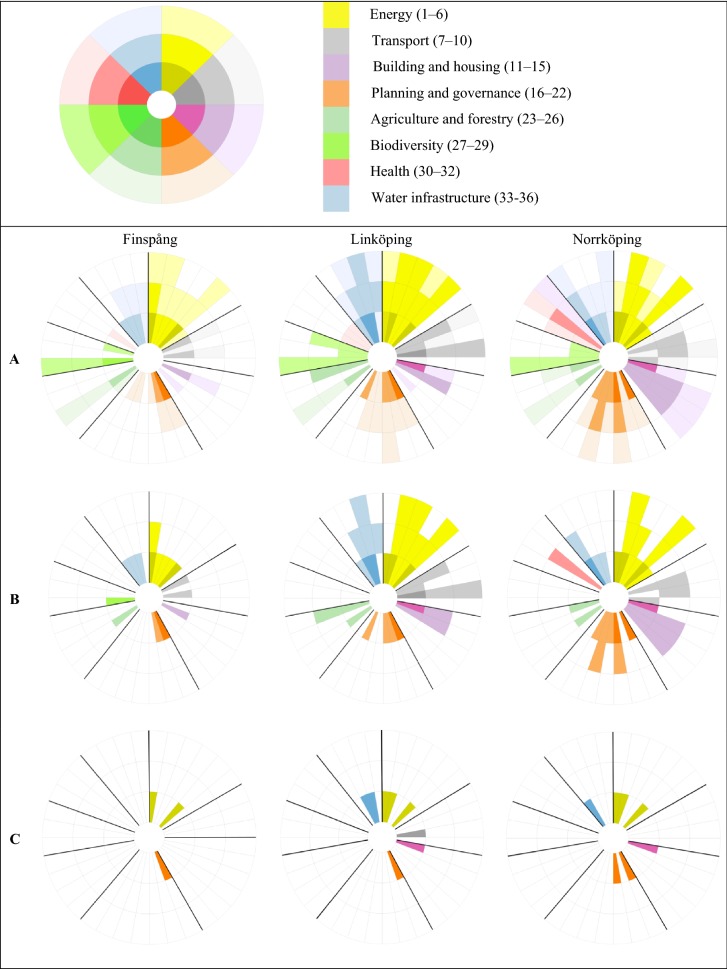
Visual representations of **a** overall UCT process progression, **b** internal spread, i.e., UCT progression is reaching the municipal organization and **c** external spread, i.e., UCT progression is also reaching relevant non-municipal actors in the three cities. N.B. The color shades represent how far the transition has progressed within each process progression phase: innovation (inner circle), experimenting (middle circle), and scaling-up (outer circle) and key activity

The combination of document study and interviews with strategically selected officials provided sufficient material to produce the visual UCT representations. The representations display common and distinct patterns of UCT process progress in the cities, clearly indicating that none of the councils pay attention to biodiversity and health, but focus far more on energy transitions (Fig. 2).

For Finspång, the diagram (Fig. 2a) demonstrates a clear dominance of activity in the energy area. This is expected, considering a long tradition of cooperating with power-intensive industry, such as Siemens Industrial Turbomachinery, and established networks on energy efficiency. Climate action has also since long been incorporated in Swedish municipal energy policy (Fenton et al. 2015). The furthest progression was found for the activity “Support energy saving among individuals and companies,” where most external actors were targeted by goals and responses. Partly this activity is prescribed in national policy, explaining its priority. However, Finspång has progressed beyond what is required by initiating energy efficiency campaigns explicitly targeting companies. Goals and plans were also developed in the water and planning areas, whereas the issue of climate transition is merely raised in other areas.

For Linköping (Fig. 2a), climate activity has also progressed farthest in the energy area, where innovating and scaling-up internally are underway. For instance, activities of the municipally owned power utility are fossil-neutral and the heating and cooling system is widely extended. This can be described as a utility-led climate transition, facilitated by the utility’s vast economic returns and high capacity and a long-lasting collaboration between the utility and engineering researchers at Linköping University. Other areas indicating high progression include “Securing emergency water supply” and “Increasing the share of public transportation, biking and walking.” Here Linköping has targeted actors across and outside the municipal administration and implemented physical responses such as bicycle routes and establishing new waterworks. Adaptation action, however, has still to progress the initiation step. Here political recognition is yet lacking.

For Norrköping (Fig. 2a), climate activity has progressed more evenly across the areas and among mitigation and adaptation actions. Activities in the energy, planning, building, and water areas suggest that UCT is underway. In the health and transport areas, activity also indicates that transition has been initiated and is beginning to progress, especially in climate adaptation and mitigation in the recent comprehensive plan, physical responses to adapt elderly care, preschool activities and buildings to heat stress, and policy measures to reduce emissions in constructions. Adaptation activity in urban storm water management is also progressing because of political guidance and experience of flooding, while progression is lower in the “Securing emergency water supply” and “Reduce GHG emission from transport” than in Linköping.

The visual representations also indicate whether actions have been spread across sectors within the internal administration (Fig. 2b). In Finspång, internal spread has only progressed to initiating, i.e., plans and goals, not to innovating and scaling-up. The internal spread of Linköping’s climate action has progressed to energy, transport, water infrastructure, and building areas. In Norrköping, climate actions have progressed internally in six areas: all except the biodiversity and agriculture and forestry areas; farthest in the energy, planning, and building areas.

In terms of external spread (Fig. 2c), the visual UCT representations demonstrate very limited progression to action targeting external actors such as the private sector and civil society. Even for initiating, few plans and goals consider external actors. This suggests that, as of yet, UCT does neither span “all” mitigation and adaptation activities nor all actors needed for enabling more systematic climate responses. External spread, thus, is a likely necessary next step to further UCT progression through strategic climate governance.

### Local evaluations of the assessment framework

The analysis of transcripts largely confirms the lack of systematic, holistic approaches to governing UCT, as highlighted in previous literature (e.g., Wamsler et al. 2014; Lee and Painter 2015). Generally, stakeholders possessed vast but narrow knowledge on specific climate activities. The municipal councillors contended that the current limited knowledge and insight about climate actions in areas where they are not active constitute a cognitive barrier for gaining a broader overview, as illustrated by a councillor:

“Our knowledge into these issues [climate transition] is probably not very high, and if we don’t really get it, it’s probably not so easy to spread. We need to find a pedagogical entrance to understand it.”

Even though all three municipalities have employed officials mandated to coordinate climate change mitigation and adaptation issues, none of them were yet using any system for comparing and analyzing action or progress across and beyond departments. This signifies limited capacity to overview UCT (Wolfram 2018; Borgström Under review), and a missed opportunity for embedding transformative capacity.

Both local climate coordinators and municipal councillors linked overviewing, i.e., grasping the overall picture, to prioritization. Without a solid overview, they found it hard to motivate more action in one area at the expense of action in another. To enable well-grounded prioritization, and cooperation, more standardized or systematic ways of comparing outcomes were considered essential. Specifically, the climate coordinator in Finspång highlighted the need for strong political leadership during the initiating phase, before officials will open up their defined tasks and initiate activity. Arguably, currently action only gets prioritized when there is a champion within a specific department that translates the fuzzy concepts into clear actions:

“The problem with this issue [climate change] is that it often depends on specific persons, it lacks a clear structure”

When presented with the visual UCT representations, the climate coordinators and municipal councillors were asked how they would interpret the image and whether they found the representation of municipal climate action adequate. Although none of them claimed to have a complete overview of their climate actions, they recognized that transition had progressed farthest in the energy area, particularly for reducing emissions. Notably, stakeholders across all cases indicated that the 36 key activities effectively captured their current climate action, and thus no additional activities were proposed. While several stakeholders expressed discontent with the low spread of many activities, no one voiced concern regarding our scoring procedure.

The visual UCT representation prompted relevant discussions on process progression. For example, Norrköping municipal councillors discussed the need for prioritizing among activities and measures to further advance UCT progression. Particularly, they discussed whether to pursue more comprehensive energy efficiency measures when the municipality’s energy use already is low carbon, or measures to reduce traffic emissions. Likewise, they compared whether artificial shading, district cooling, or planting trees were most efficient for lowering temperature.

The assessment framework revealed how obtaining an overview and a productive baseline regarding the status of current climate action is valued by municipal officers. The UCT representations further enabled debate on what responses are key for process progression in the different areas, spurring reflections about potential trade-offs, synergies, and conflicts between them. Arguably this could support learning, though the assessment framework does not explicitly consider such interactions.

The limited external spread spurred discussions on how to support agency among private and civil society actors by redirecting focus in the local climate governance. The stakeholders contended that measures reaching out in new ways to citizens and companies are needed. Involving external actors, however, was perceived as challenging, as one councillor expressed:

“This must be the hardest step to reach, but we must get there to get a real change. So, it’s a bit sad to see this picture.”

The UCT representations were thus used as a means for initiating discussions on how to better reach external actors. Initially, municipal councillors were perceiving farmers and forest owners as outside its mandate. But as the discussion evolved, the councillors described farmers and forest owners as groups that could be targeted by new municipal policy and responses. Further, one municipal councillor emphasized that due to land ownership, the municipality itself is both a farmer and a forest owner. This demonstrated that the visual representations can provoke discussions, clarifying opportunities for future actions, mainstreaming activities, and illustrating how to target community empowerment. Interestingly, this also scrutinized a key issue in urban transition, namely the role of local government in UCT processes, and more specifically, how to spur engagement and support private actor implementation (Wolfram et al. 2017).

The analysis of the transcripts also established that stakeholders found the visual representations valuable for progression and systematization. The merit of clear progression was captured by a municipal councillor stating that it became “very clear where we need to go.” They also found that the grouping of key activities into a manageable number of areas facilitated their understanding of UCT as a system. This was found particularly beneficial for areas where the stakeholders perceive themselves as non-experts, which as noted above concerns most areas.

### Suggested improvements

The stakeholders also asserted that the assessment framework could better represent UCT advancement, both in terms of measuring effects and target achievement. Measuring effects of responses were perceived as needed to visualize how much a particular activity supports UCT progression, i.e., how much emissions or climate vulnerability are reduced. Measuring effects was also linked to an experienced need for metrics on progress evaluation as argued by a stakeholder in Norrköping:

“What is measured here is the degree of attention given to this specific activity and how much we have succeeded on spreading it to as many as possible, not the measures’ effectiveness.”

Regarding such effectiveness, the municipal councillors exemplified that a huge investment in new high-speed railway represents a cross-cutting and large-scale measure intended to cause modal shifts in the whole municipal and peri-regional transport system, which the UCT representations arguably could not adequately represent. Measuring effectiveness is an often-stressed challenge in mitigation and adaptation studies, which becomes even more challenging for transition or transformative actions that influence more than one key activity of area (McCormick et al. 2013).

The municipal councillors further contended that comparative analyses of progress across sectors, especially ratios, could support prioritization of responses. By comparing across a wider array of activities outlined in the UCT representations, though, the municipal councillors noted that their thinking of new transformative ways to govern mitigation and adaptation had been improved.

Results also highlighted the need for assessments to establish a representation of target achievement, detailing the need for transition in each area or key activity, i.e., how far the present situation is from a transformed state. In relation to transition target, interviewees also acknowledged that some key areas were more challenging but also more important for achieving UCT than others:

“To grade the effects [of policies and measures] is very interesting. Because you can do so many things, but if you are very ambitious in an area where it does not have that big effect but neglect what really influences emissions that should be shown somehow.”

We see the development of a systemic understanding of relative priorities for a given place as a key feature for UCT progression. This might point to nexus approaches rather than a fixed weighting scheme that would have to arbitrate between key areas based on fixed ratios. Yet, incorporating effects, targets, and significance is challenging and points to the pertinence of balancing local urgency based on contextual factors with scientifically grounded requirements. This contributes to the often overlooked issue on how targets should be established where studies propose that they should be derived from sustainable development indicators or national and local political goals or a combination thereof (Turnheim et al. 2015; Wittmayer et al. 2016).

To respond to the demands of the stakeholders in further development of the UCT assessment framework, there are metrics available for some key activities. Most key activities, however, lack clear-cut metrics regarding their effect. Previous research indicates that only relying on existing metrics also risks shifting activity towards them (Arnott et al. 2016), and that metrics often are insensitive to local contextual differences (Tyler et al. 2016). For significance, there is no common measuring-rod for grading key activities or areas according to significance due to the context-specific nature of UCT. Visualizing significance is far from trivial since it involves relations between key activities which are interrelated in complex ways. While it is important to emphasize that the UCT representation tested here is a simplification of this complex type of information, and should not be treated as stand-alone data representations, they certainly provided a common ground for establishing some basic interrelations and possible prioritization by illuminating different perspectives in the discussions.

### Influence of the assessment framework for building local transformative capacity

Based on the climate coordinators and municipal councillors’ discussions and the way the visual UCT representation was set up, we suggest that the framework can influence the transformative capacity of local climate governance in the following ways.

First and foremost, the UCT representation can influence the capacity of local climate governance to overview the stage of transition comprehensively, as well as for the various climate activities. The climate coordinators and municipal councillors were lacking management systems and frequently stated that their current inability to overview UCT processes is a pertinent factor constraining local climate governance (c.f. McCormick et al. 2013). Of note, the identifiable differences regarding process progression also initiated further discussions of what types of responses were needed during a particular process progression phase, most notably the need for more responses targeting non-municipal actors. Overviewing the current state, hence, appears not only to advance the system awareness by building collective analysis capabilities and routines (Wolfram 2016), but also foster intense discussions on how different activities were related to one another, i.e., system dynamics (Hölscher et al. 2018).

Second, the overview also enabled a strategic discussion about transformative approaches to climate change, which the climate coordinators’ and municipal councillors were currently lacking. This indicates an improved capacity to comprehend UCT as governable and, consequently, as something that politicians could engage in. The visual representations of current UCT patterns were also regarded as easier to track. Indeed, the representations provoked discussions regarding prioritization among UCT activities, which resulted in discussing the need for initiating climate action in currently non-prioritized areas, and how to shift balance among climate responses currently underway. Also, ways to highlight the most important activities for UCT progression in a specific location were requested. These could entail large-scale responses influencing several key areas including investments in entire transport infrastructures or more intense municipal–academic partnerships (Keeler et al. 2018; Souza et al. Under review). These points all illustrate that the UCT representation motivated stakeholders to consider a wider range of governance modes (Wolfram 2016; Pahl-Wostl 2017; Hölscher et al. 2018), which would serve as a prerequisite for finding new forms of governance.

Third, by providing a common reference point for current climate activities, the UCT representations were viewed as a good basis for monitoring and following-up how the activities in any of the eight areas were progressing over time. This indicates an improved capacity for monitoring progress (Hölscher et al. 2018). To further improve this capacity, however, the climate coordinators and municipal councillors called for more specific metrics to measure the effectiveness of any specific climate activity and its significance for target achievement. Such metrics would likely be useful, but would require further research.

Fourth, through its set-up, the UCT representation explicitly conveys information on the municipalities’ experimentation, i.e., activities used to identify new measures, services, guidelines and routines, and up-scaling, i.e., mainstreaming new guidelines and routines. The evaluation system assigns a higher score when an experiment is turned into normal procedure and when it covers non-municipal actors (if applicable). This indicates a potential of the UCT assessment framework to display benefits of experimentation and up-scaling, which could facilitate innovation (Wolfram 2016; Hölscher et al. 2018).

Moreover, by explicitly suggesting incorporation of non-municipal actors in all phases of UCT progression, the framework provides an entry-point for engaging and empowering stakeholders (Wolfram 2016; Hölscher et al. 2018), though not providing explicit information on how to do this. In addition, by covering a wide range of activities, the UCT representation facilitates identification of actors in the agricultural, forestry, and tourism sectors, who were previously rarely considered as important for local climate governance. Previous studies of local climate action in Sweden have noted a lack of engagement with citizens and private sector actors (Fenton et al. 2015; Hrelja et al. 2015). The climate coordinators and municipal councillors clearly spotted the lack of targeting of non-municipal actors, resulting in a reflection over this omission. By covering a wide range of activities, the UCT representation revealed inaction within the agricultural, forestry, and biodiversity sectors (Castán Broto et al. 2018).

## Conclusions

This study set out to evaluate whether and how assessments and visualization of urban climate transition (UCT) processes can influence transformative capacity in local climate governance. Informed by literature surveys, an assessment framework was developed covering 36 key activities to clarify the breadth and contents of UCT, and process progression by outlining sets of indicators in three process progression phases: initiation, innovation, and up-scaling and by assessing whether action is spread to internal and/or external actors.

Generally, the framework worked well to represent UCT in the three cities. The structure facilitated data collection and systematization, and resulted in adequate representations of how far a city’s UCT has progressed. The Florence Nightingale chart visualization technique, used to transparently convey an overview of current progression, proved efficient, both for representing action and inaction. However, it lacks detail in presenting the type of UCT responses implemented. By being designed for highlighting progression of specific key activities, the framework does not specifically target interactions between activities. The framework, however, provided a common ground for enabling discussions on some of these basic relations, and how to prioritize based on this.

When applying the assessment framework to the climate activity in the three cities, the resulting UCT representations did capture common patterns, such as the dominance of energy-related activities (Fenton et al. 2015) and relative inaction within agricultural, forestry, and biodiversity sectors (Castán Broto et al. 2018). It was also evident that current climate actions rarely reach actors outside the municipal organization in the analyzed cases (Hrelja et al. 2015). Nevertheless, certain activity patterns within Finspång, Linköping, and Norrköping did differ, indicating an energy transition, a utility-driven transition and a more comprehensive, evenly spread pattern that has just passed initiation, respectively. The visual representations were found to capture these differences sufficiently well, despite the lack of detail, suggesting that the UCT framework could allow for comparisons between areas within and between cities.

The study finds that the UCT representation contributes to transformative capacity in local climate governance directly through developing an overview of the scope of UCT and how the transition process evolves, which also provides a basis for monitoring and following-up. This overview is viewed to make UCT more governable, which indirectly could spur local leadership. Indirectly, the UCT representation also contributes to transformative capacity through challenging what currently is considered as climate governance, who this concerns, and what types of responses are needed, i.e., fostering new forms of governance and affecting the prospects of enhancing inclusiveness. Through explicitly distinguishing between experimentation, mainstreaming, and scaling-up in its set-up, the UCT representation could potentially enhance these capacities in local climate governance. The study, however, was unable to demonstrate any such direct link. Further, the study could not detect that the UCT representation enhanced the capacity to establish a shared vision. A clearer representation of the target of the transition could be considered in future developments of the UCT assessment framework.

Since the combination of key activities, process progression indicators, and visualization technique resulted in UCT representations that were perceived as easy to understand and providing an overview of key activities, we contend that the assessment framework presented here could also be useful in the wider governance of urban sustainability transitions, and for assessing cities’ progress towards the UN Sustainable Development Goals. This will require further empirical research into key activities and process evolvement steps, potentially highlighting more complex actor interactions.

## Electronic supplementary material

Below is the link to the electronic supplementary material.

Supplementary material 1 (PDF 845 kb)
