# Supplementary material for: Developing transformative capacity through systematic assessments and visualization of urban climate transitions
Source: Ambio. 2018 Nov 3;48(5):515–28. doi: 10.1007/s13280-018-1109-9 (PMC6462282; doi:10.1007/s13280-018-1109-9)
Supplement: Supplementary file 1 — Supplementary material 1 (PDF 845 kb) [file 13280_2018_1109_MOESM1_ESM.pdf]

Ambio

Electronic Supplementary Material (ESM)

This supplementary material has not been peer reviewed.

Title: **Developing transformative capacity through systematic assessments and visualization of urban climate transitions**

Authors: **Erik Glaas, Mattias Hjerpe, Sofie Storbjörk, Tina-Simone Neset, Anna Bohman, Prithiviraj Muthumanickam, Jimmy Johansson**

Table S1. Combinations of search words for identifying articles containing key UCT activities.

**Search words. climate change +**	**Found articles**
transition + adaptation + city	44
transition + adaptation + municipality	12
transformation + adaptation + municipality	16
transformation + adaptation + city	57
transformation + mitigation + city	31
transformation + mitigation + municipality	11
transition + mitigation + municipality	6
transition + mitigation + city	24
**Total**	**201**

Table S2. Scores of UCT process progression for the 36 key activities in Finspång. The results are displayed in the left column of figures 2A-C in the article.

UCT activity	Initiation (inner circles)	S1	Innovating (middle circles)	S2	Scaling-up (outer circles)	S3
**Energy**						
1. Support energy saving among individuals and companies	**Plan:** Targets for energy efficiency services<br>**Investigation**: Assess companies with high use of fossil energy to target energy saving services	**3**	**Service:** Free energy saving advice to households, companies and org.<br>**Service**: Lectures about and help to identify energy efficiency measures for companies	**2**	**New procedure:** Targeting 5 new companies/year to find new measures	**1**
2. Optimize waste management	**Plan**: Waste Management Plan 2014-2018<br>**Goal:** Decrease the amount of waste from municipal activities with 25% by 2018<br>**Goal:** Decrease the amount of household waste	**2**	**Guideline**: Provide information about recycling of building material in conjunction with all building and demolition permits	**1**	**New procedure**: All burnable municipal waste and imported waste used to produce district heating	**1**
3. Decrease the use of non-renewable energy	**Goal:** Decrease CO_2 emissions with 40% by 2020 compared to 1990<br>**Plan**: Energy and climate strategy 2015-2018<br>**Goal**: Decrease energy consumption with 20% by 2020 compared to 2008<br>**Goal:** The municipal organization should be fossil free by 2025	**2**	**Guideline:** Only buy renewable energy hydro power in public operations<br>**Response:** Systematically replace oil to renewable alternatives in the district heating system	**1**		**0**
4. Increase the share of renewable energy	**Goal**: Fossil free energy use in the public sector by 2025<br>**Goal**: At least 50% renewable energy in transport and heating by 2020<br>**Goal**: At least 5% of the total used electricity should be own produced renewable energy by 2020<br>**Investigation**: Assess potentials for solar panels on official buildings<br>**Investigation:** Wind power assessment and plan	**2**	**Experiment:** Developing a biogas plant from sludge to produce electricity and heat<br>**Response:** Decrease the use of fossil fuels in district heating<br>**Service**: Information to citizens on installing solar power<br>**Experiment:** Installing a few solar panels on official buildings	**1**		**0**
5. Develop effective district heating and cooling systems	**Investigation:** Assess possibilities to use more waste heat from industry in the district heating system<br>**Goal:** Fossil free district heating and cooling by 2020	**3**	**Response**: Using industrial excess heat (SSAB) for district heating<br>**Response**: Changing to bio oil in reserve power stations	**1**	**New procedure:** All burnable municipal waste used to produce district heating and electricity	**1**
6. Adaptation of energy system, grid and IT	**Investigation**: Assessment of reserve power needs for Community-critical infrastructure such as elderly care/health care<br>**Issue raised:** Adaptation of energy grid	**1**	**Response:** Further replacement of overhead transmission lines to underground cables.	**0**		**0**

UCT activity	Initiation (inner circles)	S1	Innovating (middle circles)	S2	Scaling-up (outer circles)	S3
**Transport**						
7. Reduce GHG emissions from passenger transports.	**Goal:** Decrease CO2 emissions with 40% by 2020 compared to 1990<br>**Goal**: All owned and leased municipal vehicle should be fossil free by 2025<br>**Goal:** At least 50% renewable energy in transport and heating by 2020<br>**Investigation:** Assess potential for increasing the use of electric cars by developing charging infrastructure<br>**Investigation:** Assess potential for biodiesel in municipal cars	**2**	**Guideline**: All new municipal vehicles shall be fossil free<br>**Service**: Information to citizens on low-carbon traveling<br>**Experiment:** Installing charging stations for electric cars<br>**Experiment:** Run busses on biodiesel	**1**		**0**
8. Decrease GHG emissions from goods transports	**Goal**: 75 % of all waste collection transports should be run by renewable fuels by 2018<br>**Issue raised:** Transfer more goods transportation from trucks to rail	**1**		**0**		**0**
9. Increase the share of public transportation, biking and walking	**Investigation:** Assess possibilities to increase public transportation, biking and walking among inhabitants<br>**Goal**: Plan the municipality to make biking, walking and public transportation more attractive<br>**Goal:** Increase public transports<br>**Plan:** Program for walking and biking	**2**	**Response:** Building new/developing walking and biking routes and new traffic signs<br>**Experiment:** Municipal employees can try bus cars<br>**Service:** information campaigns on biking	**1**		**0**
10. Adaptation of roads and transport infrastructure		**0**		**0**		**0**
**Building and housing**						
11. Support sustainable land use through urban densification		**0**		**0**		**0**
12. Increase energy efficiency in buildings	**Goal:** Decrease energy use with 20% by 2020 compared with 2008<br>**Goal**: Energy use in public buildings should be maximum 140 kWh/m2<br>**Investigation:** Assess possibilities for energy efficiency measures in public buildings	**2**	**Response**: Change all fossil heating to non-fossil in public buildings<br>**Response**: Energy efficiency measures taken in the municipal housing company Vallonbygden	**1**		**0**
13. Decrease emissions from constructions		**0**		**0**		**0**
14. Adaptation of official buildings and information to private house owners	**Goal:** Ensure reliable electricity to Community-critical activities by 2020<br>**Investigation**: Assess reserve power needs for Community-critical activities such as elderly care/health care	**1**		**0**		**0**
15. Adaptation of cultural heritage (e.g. buildings with cultural values)		**0**		**0**		**0**

UCT activity	Initiation (inner circles)	S1	Innovating (middle circles)	S2	Scaling-up (outer circles)	S3
**Planning and governance**						
16. Mitigation considerations inherent in urban planning	**Goal:** Decrease CO_2 emissions with 40% by 2020 compared to 1990<br>**Issue raised:** Considerations to mitigation in holistic plan<br>**Issue raised:** New rural development areas should be planned in public transportation paths	**3**	**Guideline:** Guiding principles and sections in master plan	**1**		**0**
17. Cooperation with citizens and companies for resilience and low GHG	**Goal:** Engage in active dialog about environmental issues with citizens, authorities, universities and companies	**2**	**Experiment:** Climate advisor has initiated dialogs with companies	**1**		**0**
18. Adaptation considerations inherent in urban planning	**Issue raised:** Considerations of adaptation in holistic plan<br>**Goal:** Develop a municipal adaptation strategy	**1**		**0**		**0**
19. Increase share of green-blue infrastructure		**0**		**0**		**0**
20. Holistic flood risk management	**Issue raised:** Flood risks discussed in comprehensive plan	**1**		**0**		**0**
21. Inter-municipal cooperation and learning for resilience and low GHG	**Cooperation:** Part of the adaptation and mitigation networks “Covenant of Mayors” (international), “the Climate municipalities” (National) and regional energy and climate network	**1**		**0**		**0**
22. Adaptation of tourism in a changing climate		**0**		**0**		**0**
**Agriculture and forestry**						
23. Decrease GHG emissions from agriculture and forestry	**Issue raised:** In comprehensive plan	**1**		**0**		**0**
24. Enhance usage of locally produced food and timber	**Goal:** Increase the share of locally produced, organic food and decrease meet consumption (SMART)	**2**	**Response:** Public procurements according to SMART-principle	**1**	**New procedure:** SMART-principle spread internally	**1**
25. Adaptation of agriculture and forestry on own land or info. to producers		**0**		**0**		**0**
26. Facilitate urban and peri-urban agriculture and gardening		**0**		**0**		**0**

UCT activity	Initiation (inner circles)	S1	Innovating (middle circles)	S2	Scaling-up (outer circles)	S3
**Biodiversity**						
27. Increase the share of organic food (schools, health care)	**Plan:** Diet and food policy<br>**Goal**: Decrease food waste in municipal facilities (incl. schools)<br>**Goal:** Increase the share of locally produced, organic food and decrease meet consumption (SMART)<br>**Goal:** The share of organic food in municipal procurements should be at least 31%	**2**	**Response:** Public procurements according to SMART-principle	**1**	**Internal spread:** Reached 25% organic food in public facilities	**1**
28. Mainstream ecosystem-based adaptation in environmental management		**0**		**0**		**0**
29. Preserve biological diversity in a changing climate	**Issue raised**<br>**Goal:** Preserve biological diversity	**1**		**0**		**0**
**Health**						
30. Identify vulnerable groups (for heat, flooding, etc.)		**0**		**0**		**0**
31. Adaptation to avoid health related impacts (for heat, flooding, etc.)	**Investigation**: Assess reserve power needs for Community-critical activities such as elderly care/health care	**1**		**0**		**0**
32. Adapt management practices in health- and social care		**0**		**0**		**0**
**Water infrastructure**						
33. Assess vulnerability of and adapt urban storm and waste water systems	**Plan:** Water and sanitation strategy<br>**Goal:** Consider and adapt Water and sanitation systems to a changing climate<br>**Cooperation:** Establish a cross departmental storm water management group<br>**Cooperation:** Establish cooperation with neighboring municipalities	**2**		**0**		**0**
34. Assess vulnerability of and adapt drinking water systems	**Plan**: Water and sanitation strategy<br>**Goal**: Consider and adapt Water and sanitation systems to a changing climate	**2**	**Response:** Developed water protection of ground water in Byle, Grytgöl, Hävla och Igelfors	**1**		**0**
35. Secure reserve water (in case of e.g. drought or contamination)	**Plan:** Water and sanitation strategy<br>**Goal:** Secure and ensure present and future drinking water needs	**2**	**Response:** Developing two new water protection areas (Hunn and Rejmyre)	**1**		**0**
36. Decrease leakage in water infrastructure	**Plan:** Water and sanitation strategy	**1**	**Response:** Ongoing renewal of pipes	**1**		**0**

Table S3. Scores of UCT process progression for the 36 key activities in Linköping. The results are displayed in the middle column of figures 2A-C in the article.

UCT activity	Initiation (inner circles)	S1	Innovating (middle circles)	S2	Scaling-up (outer circles)	S3
**Energy**						
1. Support energy saving among individuals and companies	**Plan:** Targets for energy efficiency services	**3**	**Service**: Free energy saving advice to households, companies and organisations<br>**Service**: Information material and contact persons	**2**	**New procedure.** Targeted information on installing solar power among households	**1**
2. Optimize waste management	**Goal**: Use waste for heat and electricity production<br>**Goal**: Waste levels should be minimized<br>**Goal**: food waste should be used for biogas production	**3**	**Response:** Collecting public and private food waste for biogas production (green bag)<br>**Response:** Burning waste to produce heat and electricity	**2**	**New procedure**: Production of biogas from all food waste<br>**New procedure**: Developed infrastructure for biogas for fuel<br>**Response:** All waste, except food waste, and imported wastee used to produce heat and electricity	**2**
3. Decrease the use of non-renewable energy	**Goal:** being fossil free by 2025, agreement across political parties<br>**Plan:** Climate and energy plan under way	**2**	**Response**: Change street lights to LED ongoing<br>**Guideline:** All public departments should develop mitigation strategies	**2**	**New procedure**: Mitigation included in overall budget<br>**New procedure**: Only renewable energy in public buildings<br>**New procedure**: The municipal real estate company Lejonfastigheter uses only fossil free electricity	**2**
4. Increase the share of renewable energy	**Goal:** increase the share of renewable energy<br>**Goal:** increase production and infrastructure for biogas	**2**	**Experiment**: In short term over-produce climate neutral electricity to sell (burning imported waste)<br>**Service**: Encourage and guide instalment of private solar energy in buildings<br>**Service:** Information campaigns to increase the development of solar energy<br>**Response:** Increased production of municipal wind and solar power	**2**	**New procedure**: Investment funds for fossil free energy production<br>**New procedure**: The municipal real estate company Lejonfastigheter uses only fossil free electricity	**1**
5. Develop effective district heating and cooling systems	**Goal:** District heating shall be the main source of heating	**3**	**Response:** Expanding existing district heating and cooling systems	**2**	**New procedure:** District heating and cooling run by burning waste	**2**
6. Adaptation of energy system, grid and IT		**0**		**0**		**0**

**UCT activity**	**Initiation (inner circles)**	**S1**	**Innovating (middle circles)**	**S2**	**Scaling-up (outer circles)**	**S3**
**Transport**						
7. Decrease GHG emissions from passenger transports	**Goal:** Fossil free by 2025, agreement across political parties<br>**Goal**: All buses should be run by fossil free fuel<br>**Goal**: Increase the cost for car parking<br>**Plan:** Traffic strategy 2010	**2**	**Response**: Procurement of biogas driven car pool for public and private use<br>**Response**: Lowered speed limits<br>**Response**: Public GHG compensation system for travels<br>**Response**: Investments in fossil free cars for public departments<br>**Response**: Increase the number of charging station for electric cars and develop car pools	**2**	**New procedure**: All public transportation is fossil free	**1**
8. Decrease GHG emissions from goods transports	**Goal:** Increase the share of goods transportation on rail<br>**Investigation:** Conducted a pre-study on coordinated goods transportation system for public goods	**2**	**Response**: Financed a coordinated goods transportation system for public goods<br>**Response:** Plan for decreasing amount of goods transportation in public administration	**1**		**0**
9. Increase the share of public transportation, biking and walking	**Goal**: Becoming the leading biking city in Europe<br>**Plan:** Bicycle plan<br>**Goal:** Increase share of bicycle travel from 30% to 40%<br>**Goal**: Increase public transportation to 20% of all travels<br>**Goal:** Build a new more effective central railway station<br>**Goal**: Decrease the share of car traffic from 60% to 40%	**3**	**Response:** Expand biking and walking routs<br>**Response:** Building parking lots to facilitate bus and train commuting<br>**Service:** Public information campaigns on biking<br>**Response:** Increase the number of bicycle parking's<br>**Guideline:** Bicycle planning are given priority in traffic planning<br>**Response**: Plan to increase commuting among public staff	**2**	**New procedure**: Ongoing developments of bike and walking paths	**2**
10. Adaptation of roads and transport infrastructure	**Investigation:** Assessed risks for flooding on roads and transport from 100-year rain	**1**		**0**		**0**
**Building and housing**						
11. Support sustainable land use through urban densification	**Goal**: Densify the city centre by lowering the amount of cars<br>**Plan**: Strategy to make the city denser	**3**	**Response:** Most of new housing close to city centre<br>**Experiment**: New city district Vallastaden – Free elevation, vertical green area	**2**		**0**
12. Increase energy efficiency in buildings	**Goal:** Fossil free by 2025, agreement across political parties	**2**	**Response:** Energy efficiency measures in public buildings<br>**Service**: Information campaigns to citizens on energy efficient houses	**2**		**0**
13. Decrease emissions from constructions		**0**		**0**		**0**
14. Adaptation of official buildings and information to private house owners	**Goal**: No community-critical infrastructure should be built within 1000-year flood areas	**1**		**0**		**0**
15. Adaptation of cultural heritage (e.g. buildings with cultural values)	**Goal:** Consider and assess cultural values in in areas for new development	**0**		**0**		**0**

UCT activity	Initiation (inner circles)	S1	Innovating (middle circles)	S2	Scaling-up (outer circles)	S3
**Planning and governance**						
16. Mitigation considerations inherent in urban planning	**Goal:** Fossil free by 2025, agreement across political parties<br>**Goal**: All municipal planning should consider climate change mitigation<br>**Cooperation:** Joint climate vision with Norrköping municipality	**3**	**Guidelines:** Principles and sections in Comprehensive plans	**1**		**0**
17. Cooperation with citizens and companies for resilience and low GHG	**Goal**: Increase the engagement about climate change issues among citizens	**2**	**Service:** Information projects on a climate friendly lifestyle.<br>**Service:** Municipal Climate Advisor.	**1**		**0**
18. Adaptation considerations inherent in urban planning	**Goal:** All municipal planning should consider climate change adaptation<br>**Goal:** No new buildings should be built within 1000-year flood areas<br>**Plan:** Decision taken to develop an adaptation plan in the municipality	**2**	**Guideline:** Building in risk zones for land-slides demands specific analyses	**1**	**New procedure:** All new plans considers flood risks	**1**
19. Increase share of green-blue infrastructure	**Goal**: Parks and green areas with high biological values should be further developed	**1**	Blue infrastructure is raised, but no experiments or responses taken.	**1**		**0**
20. Holistic flood risk management	**Investigation:** Risk maps of flooding from watercourses have been developed	**1**	Issue raised.	**1**		**0**
21. Inter-municipal cooperation and learning for resilience and low GHG	**Cooperation:** Part of the adaptation and mitigation networks “Covenant of Mayors” (international) and “the Climate municipalities” (National). Joint climate vision with Norrköping	**2**		**0**		**0**
22. Adaptation of tourism in a changing climate		**0**		**0**		**0**
**Agriculture and forestry**						
23. Decrease GHG emissions from agriculture and forestry	**Goal**: Increase the share of ecological farming on municipal land	**0**		**0**		**0**
24. Enhance usage of locally produced food and timber	**Goal:** Use locally produced and organic food as much as possible	**2**	**Response:** More buildings built with local timber	**1**	**New procedure:** Possibilities for building with timber systematically evaluated	**1**
25. Adaptation of agriculture and forestry on own land or info. to producers		**0**		**0**		**0**
26. Facilitate urban and peri-urban agriculture and gardening	**Goal**: Preserve and develop allotments near the city centre	**2**	**Experiment:** New areas for small-scale urban gardening on municipality land have been started in 5 city areas	**2**		**0**

**UCT activity**	**Initiation (inner circles)**	**S1**	**Innovating (middle circles)**	**S2**	**Scaling-up (outer circles)**	**S3**
**Biodiversity**						
27. Increase the share of organic food (schools, health care)	**Goal**: Environmental and climate criteria in public procurement	1	**Response:** Fairtrade City diploma increase the share of Fairtrade food in schools, health care, etc.	1		1
28. Mainstream ecosystem-based adaptation in environmental management	**Issue raised**<br>**Goal**: Develop green areas in the city for biodiversity, flood management and recreation	1		0		0
29. Preserve biological diversity in a changing climate	**Goal**: improve possibilities for biodiversity in parks<br>**Investigation:** increase knowledge and management related to invasive species on land and in water	1	**Response:** Measures to facilitate urban biodiversity through building dams and wetlands, develop nature preserves, arrange controlled forest burns, tree planting, greed areas and urban grazing<br>**Service:** Facilitate for citizens to explore nature through information, learning, new walking paths, guiding, etc.<br>**Service:** Inform land owners of how construction and building affect biodiversity	1		0
**Health**						
30. Identify vulnerable groups (for heat, flooding, etc.)	**Issue raised:** The municipal risk and vulnerability analysis acknowledges further analysis of risks from heat waves.	1		0		0
31. Adaptation to avoid health related impacts	**Issue raised:** The municipal risk and vulnerability analysis acknowledges further analysis of risks from heat waves.	1		0		0
32. Adapt management practices in health- and social care	**Issue raised:** The municipal risk and vulnerability analysis acknowledges further analysis of risks from heat waves.	1		0		0
**Water infrastructure**						
33. Assess vulnerability of and adapt urban storm and waste water systems	**Goal:** Improve storm water treatment<br>**Goal:** The amount of green roofs shall increase<br>**Goal:** Increase the amount of open storm water solutions<br>**Investigation:** Assessed risk of flooding from 100-year rain<br>**Plan:** Storm water policy, strategy and plan under development<br>**Plan:** Strategy on flood surface areas in new developments	2	**Service:** Information to landowners on how to take care of more rain-water on their land	1		0
34. Assess vulnerability of and adapt drinking water systems	**Goal:** Drinking water use shall decrease and the water be safe<br>**Investigation:** Vulnerability assessment conducted	3	**Response:** Recovery plan developed	2	**New procedure:** Recovery plan in place and followed up internally	1
35. Secure reserve water (in case of e.g. drought or contamination)	**Investigation:** Assessment of potentials for reserve water for all inhabitants	3	**Response:** An emergency water source with ground water is located to Slaka<br>**Response:** The designated reserve water supply source is being developed into a protected area<br>**Response:** Two remote waterworks are being redeveloped to support inhabitants in the municipality with emergency water supply<br>**Response:** Funding for redeveloping the two emergency waterworks<br>**Response:** Four further water protection areas are being developed	2	**New Procedure:** All inhabitants can get emergency water for drinking (5l/day)	2
36. Decrease leakage in water infrastructure	**Issue raised**: Issue included in water strategy	1	**Response:** Development of a tool to measure and decrease leakage in drinking water pipes<br>**Response:** Recovery plan developed	2	**New Procedure:** Continuous investigations	1

Table S4. Scores of UCT process progression for the 36 key activities in Norrköping. The results are displayed in the right column of figures 2A-C in the article.

UCT activity	Initiation (inner circles)	S1	Innovating (middle circles)	S2	Scaling-up (outer circles)	S3
**Energy**						
1. Support energy saving among individuals and companies	**Plan:** Targets for energy efficiency services<br>**Issue raised**: Responsibility of: Näringslivskontoret, Bygg- och miljökontoret guide agriculture. Ekonomi- och styrningskontoret work with external actors	**3**	**Experiment:** Municipal housing company HNAB trained 60-80 energy ambassadors (voluntary)<br>**Service:** Municipal climate advisor<br>**Experiment:** two seminars for SMEs	**1**		**0**
2. Optimize waste management	**Goal:** Waste levels should be minimized, no quantitative goal<br>**Plan:** Waste management plan with many measures	**3**	**Response:** Biogas from food waste<br>**Response:** Household source separation<br>**Response:** Burning waste for energy production	**2**	**New procedure:** Response fully implemented. Source separation implemented. Biogas from food waste implemented.	**2**
3. Decrease the use of non-renewable energy	**Goal:** Only renewable energy in all municipal activities 2030. Municipal organizations clearly assigned responsibility in 4-year plan of action	**2**	**Response:** New LED street lightning.<br>**Experiment/Response:** Use public procurement (and agreements with contractors) to decrease GHG emissions.	**2**	**New procedure:** Responses fully implemented internally in 2016	**1**
4. Increase the share of renewable energy	**Goal:** Only renewable energy production<br>**Investigation:** Roofs suitable for solar panels<br>**Investigation:** Wind energy (but no projects under way)	**2**	**Response:** Solar panels installed in 2013 (HNAB) and 2014 (Norrevo).<br>**Response:** Electricity via biogas from wastewater.	**1**		**0**
5. Develop effective district heating and cooling systems	No goal but measure installed widely.	**3**	**Response:** Renewable fuel for heating in Händelöverket (E.ON). Cooling in place, but not extended at large.	**2**	**New procedure:** Expansion of district heating and cooling	**2**
6. Adaptation of energy system, grid and IT	**Goal:** Year 2030 the energy grid and system should be robust towards climate impacts	**2**		**0**		**0**
**Transport**						
7. Decrease GHG emissions from passenger transports	**Goal:** Municipal goal covers transport.<br>**Plan:** Measures in Climate and Energy plan. In Comprehensive plan: Car transport should not increase.<br>**Internal forum:** Working groups on transition to renewable fuels.	**1**	**Response:** Planning for a new high-speed railway and central railway station.<br>**Response:** Sustainable travelling for municipal politicians and staff.<br>**Response:** Parking norms in city centre. Parking pricing.<br>**Investigation:** Public procurement criteria for fossil free cars.	**1**		**0**
8. Decrease GHG emissions from goods transports	**Investigation:** Coordinated goods transport.<br>**Plan:** Comprehensive plans of new railway transport.	**2**	**Response:** Large-scale investment in railway transport underway.<br>**Experiment:** Coordinated goods transport, organise a distribution hub in 2016.	**2**		**0**
9. Increase the share of public transportation, biking and walking	No goal.<br>**Planning principle:** New Comprehensive plan prioritizes public transport, biking and walking over cars. Also introduces "Promenadstaden".	**1**	**Response:** Route optimisation of school transport.<br>**Response:** Plan for a biking lane net in Norrköping.<br>**Experiment:** Virtual meetings, video conferencing.	**2**	**Response:** Substantial investment in cycling lanes.	**1**
10. Adaptation of roads and transport infrastructure	No goal.<br>**Investigation:** Assessed risks for flooding on roads (e.g. 100 yr floods).<br>**Plan:** Municipal adaptation guidelines under way.	**2**	**Service:** Adaptation coordinator at the Technical (streets) department.<br>**Guideline:** Distribution of responsibilities for flooding.	**1**		**0**

UCT activity	Initiation (inner circles)	S1	Innovating (middle circles)	S2	Scaling-up (outer circles)	S3
**Building and housing**						
11. Support sustainable land use through urban densification	No goal.<br>Planning principle: New Comprehensive plan prioritizes densification and building close to existing public transport	**3**	**Response:** Most of new housing in city centre. Butängen och Inre hamnen convert industrial property to residential and commercial areas.	**1**		**0**
12. Increase energy efficiency in buildings	**Goal:** Decrease energy use by 30%, increase efficiency to 50% by 2030 (Energy plan). Municipal organisations clearly assigned responsibility in 4-year plan of action.<br>Objective to engage external actors; business office and climate and energy advisor are responsible.	**2**	**Response:** Measures are allocated to the ones with lowest energy performance/highest use.<br>**Response:** More efficient heating of municipal greenhouse.<br>**Experiment:** School kitchen (Söderport).<br>**Experiment:** Sustainability ombudsmen in kindergardens and elderly care units.<br>**Response:** Permittable to weigh in energy efficiency when adapting housing.<br>**Experiment:** Networks and seminars with SMEs.<br>**Response:** Inform public, companies and organisations about climate and energy e.g. events such as Earth Hour.	**2**	**Scaling-up:** School kitchen (half) and economic incentives.	**1**
13. Decrease emissions from constructions	No goal.<br>**Plan:** Measures proposed in Climate and Energy Action Plan.<br>**Investigation:** Strategy for implementing energy demands (procurements), unclear if this is valid for construction.	**2**	**Response:** Informs the construction companies about how to build energy efficiently and discusses energy issues when councilling with the construction company.<br>**Response:** Inform the public about energy efficient construction (those queing for lots).<br>**Response:** Tougher demands on municipality's internal construction (BBR21).<br>**Response:** Fossil free fuels in public procurement of construction	**2**	**Scaling-up:** Demands in public procurement of construction / entreprenörer.	**1**
14. Adaptation of official buildings and information to private house owners	**Goal:** "Norrköping municipality works actively to adapt the municipality to climate change." This applies to the municipal real estate utility Norrevo. Norrevo participates in the climate adaptation group.<br>**Proposed goal:** Proposal to prescribe that climate adaptation measures should be considered in all remodelling / renovation of public buildings.	**2**	**Response:** Comfort cooling in five elderly housings.<br>**Response:** Stormwater measures on elderly housing and kindergardens.<br>**Response:** Sun protection on 40 kindergardens.<br>**Response:** Increased awareness of drainage and ground water issues.<br>**Response:** Geothermal cooling heating is planned for more elderly housing.<br>**Response:** Technical department has adapted playgrounds and a new parking space.	**2**	**Scaling-up:** Sun protection on 40 more kindergardens.<br>**Scaling-up:** Comfort cooling in more elderly housings.	**1**
15. Adaptation of cultural heritage (e.g. buildings with cultural values)	No explicit consideration of cultural heritage.	**0**		**0**		**0**

UCT activity	Initiation (inner circles)	S1	Innovating (middle circles)	S2	Scaling-up (outer circles)	S3
**Planning and governance**						
16. Mitigation considerations inherent in urban planning	**Planning principles:** Energy considerations is part in comprehensive and detailed planning.<br>**Cooperation:** Joint climate vision with Linköping.<br>**Investigation:** How can the municipality pose tougher energy demands than national standards in view of legal barrier.	**3**	**Guidelines:** Principles and sections in comprehensive plans.	**1**		**0**
17. Cooperation with citizens and companies for resilience and low GHG	No goal.	**1**	**Service:** Seminars and activities targeted to the public.<br>**Service:** Municipal climate advisor (GHG mitigation).	**1**		**0**
18. Adaptation considerations inherent in urban planning	**Planning principles:** Climate adaptation in comprehensive plans. Proposal for Strategies and guidelines described in New comprehensive plan. Lowest locations and flood surfaces indicated.<br>**Internal forum:** Guidelines for climate adaptation.	**3**	**Response:** Guidelines for climate adaptation in planning.<br>**Investigation:** Requirements and analyses are needed when building in areas with flood risk.<br>**Response:** Urban planning department has developed a checklist for climate adaptation that applies to detailed plans.<br>**Response:** Lowest allowable floor level.	**2**	**Scaling-up:** Internal spread from urban planning to water utility and technical department underway.	**1**
19. Increase share of green-blue infrastructure	Initiated guidelines for ecosystem services.<br>**Plan:** Section on green-blue infrastructure in new comprehensive plan.	**2**	Guidelines underway.	**1**		**0**
20. Holistic flood risk management	No goal.<br>**Plan:** New guidelines for Sustainable Stormwater Management underway (sustainability, water quality and climate adaptation).<br>**Response:** Developed a distribution of responsibilities.<br>**Response:** Inter-departmental stormwater action plan.	**2**	**Experiment:** Hired new competence regarding open stormwater handling.<br>**Response:** Several measures in the interdepartmental action plan.<br>**Investigations:** opening up Ljura bäck	**2**	**Scaling-up:** Continue to implement open stormwater measures in residential areas	**1**
21. Inter-municipal cooperation and learning for resilience and low GHG	No goal.<br>**Cooperation:** Member of Energy agency's Uthållig kommun, Fossil free Sweden. EU-funded project future mobility in Norrköping. Joint climate vision with Linköping.	**2**	**Response:** Staff is active in national climate adaptation inquiry. Collaborates with universities and companies on both energy and climate adaptation (and SMHI). Staff active in sectoral agencies.	**1**		**0**
22. Adaptation of tourism in a changing climate	No goal. No investigation.	**0**		**0**		**0**
**Agriculture and forestry**						
23. Decrease GHG emissions from agriculture and forestry	No goal.	**0**		**0**		**0**
24. Enhance usage of locally produced food and timber	No goal.<br>**Guidelines:** Locally produced food is encouraged in public procurement (even extra funds for this). Nothing on timber.	**2**	**Response:** SMART food in kindergardens.	**1**	**Scaling-up:** SMART food in more units.	**1**
25. Adaptation of agriculture and forestry on own land or info. to producers	No goal.	**0**		**0**		**0**
26. Facilitate urban and peri-urban agriculture and gardening	**Proposed Guidelines**.	**2**	**Experiment:** City farming in parks. Collaboration with NGOs.	**1**		**0**

UCT activity	Initiation (inner circles)	S1	Innovating (middle circles)	S2	Scaling-up (outer circles)	S3
**Biodiversity**						
27. Increase the share of organic food (schools, health care)	**Plan:** Partly in guidelines (transport emissions). Use public procurement.	**1**	**Response:** SMART food in kindergardens. Very little red meat in kindergardens and schools. **Response:** Kitchen ambassadors.	**1**	**Scaling-up:** SMART food in more units.	**1**
28. Mainstream ecosystem-based adaptation in environmental management	No goal. **Initiated** developing guidelines for ecosystem services.	**1**		**0**		**0**
29. Preserve biological diversity in a changing climate	No goal.	**1**		**0**		**0**
**Health**						
30. Identify vulnerable groups (for heat, flooding, etc.)	No goal. **Investigation:** Vulnerability analysis underway.	**1**	Issue raised.	**1**		**0**
31. Adaptation to avoid health related impacts (for heat, flooding, etc.)	No goal. **Planning principle:** Design elderly housing, kindergartens and schools to avoid excessive indoor temperature in comprehensive plan.	**2**	Response: Norrevo is equipping elderly housing with cooling. Response: Sun protection in kindergardens.	**2**	**Scaling-up:** Norrevo is equipping elderly housing with cooling. **Scaling-up:** Sun protection in kindergardens (40 more in 2017).	**1**
32. Adapt management practices in health- and social care	No goal. **Guidelines:** Developed and how to distribute them.	**1**	**Response:** A checklist has been distributed to all units. **Response:** Warnings are distributed prior to heat waves (three days above 26 degrees)?	**1**	**Scaling-up:** A checklist has been distributed to all units.	**1**
**Water infrastructure**						
33. Assess vulnerability of and adapt urban storm and waste water systems	**Goal:** "Norrköping municipality works actively to adapt the municipality to climate change.". **Guidelines:** New directive on distribution of responsibilities. **Guidelines:** Revised Guidelines. **Internal forum:** Inter-departmental working group.	**3**	**Response:** Action Plan. **Investigation:** Map 100 yr flood, cloudburst (200, 500 ys). **Investigation:** Modelling water flow in urban water courses (100, 1000 ys). **Investigation:** Identified most recipients. **Response:** Works actively with adapting the stormwater and water system in "most" vulnerable areas. **Response:** Flood reservoirs are constructed and more under way. **Experiment:** Have started with open stormwater solutions (reqruited competence, built a few pilot ponds) and new playgrounds with "lowest locations". **Investigation:** stormwater fee and judicial aspects to incentivize climate adaptation.	**2**	**Scaling-up:** More flood reservoirs, SEK 150 million will be invested during ten years.	**1**
34. Assess vulnerability of and adapt drinking water systems	**Goal:** "Norrköping municipality works actively to adapt the municipality to climate change." Awareness of lacking water supply source is increasingly recognized by leading politicians. **Investigations** are made but few alternatives exist.	**2**	**Response:** Invested in better technical equipment to be able to produce drinking water even when water quality in supply source is lower.	**1**		**0**
35. Secure reserve water (in case of e.g. drought or contamination)	**Goal:** "Norrköping municipality works actively to adapt the municipality to climate change." Awareness of lacking water supply source is increasingly recognized by leading politicians. **Investigations** are made but few alternatives exist.	**2**	**Response:** Invested in better technical equipment to be able to produce drinking water even when water quality in supply source is lower.	**1**		**0**
36. Decrease leakage in water infrastructure	No goal. **Investigation:** Water utility is filming and modelling piped system	**1**	**Response:** Water utility investment in renewal of pipes	**1**	**Scaling-up:** Leakage will be decreased as a result of the SEK 150 million renewal.	**1**

Table S5. Identified key UCT activities merged into eight thematic areas

Area	Transition activities	References
Energy	1. Support energy saving among individuals and companies	Knuth (2010), Moloney and Horne (2015), and Johansson et al. (2016)
	2. Optimize waste management	Corvellec et al. (2013), Eames et al. (2013), Uyarra and Gee (2013), and Zaman and Lehmann (2013)
	3. Decrease the use of non-renewable energy	Heiskanen et al. (2011) and Moloney and Horne (2015)
	4. Increase the share of renewable energy	Busch and McCormick (2014)
	5. Develop effective district heating and cooling	Hawkey et al. (2013)
	6. Adaptation of energy system, grid, and IT	Eames et al. (2013), Rosenzweig and Solecki (2014), and Carter et al. (2015)
Transport	7. Reduce GHG emissions from passenger transports	Knuth (2010), Liu et al. (2012), Burch et al. (2015), and Shukla and Dhar (2015)
	8. Reduce GHG emissions from goods transports	Knuth (2010), Liu et al. (2012), and Oshiro and Masui (2015)
	9. Increase the share of public transportation,<br>biking, and walking	Greca et al. (2011), Mejı´a-Dugand et al. (2013), and Moloney and Horne (2015)
	10. Adaptation of roads and transport<br>infrastructure	Eames et al. (2013); Rosenzweig and Solecki (2014)
Building and housing	11. Support sustainable land use through urban densification	Neuman (2005) and La Greca et al. (2011)
	12. Increase energy efficiency in buildings	Colombier and Li (2012), Kocabas (2013), Hofmann et al. (2015), and Moloney and Horne (2015)
	13. Decrease emissions from constructions	Liu et al. (2012)
	14. Adaptation of official buildings and information to private house owners	Eames et al. (2013), Rosenzweig and Solecki (2014), and Glaas et al. (2015)
	15. Adaptation of cultural heritage (e.g.,<br>buildings with cultural values)	Davoudi et al. (2014) and Glaas et al. (2015)
Planning and governance	16. Mitigation considerations inherent in urban planning	Martinez et al. (2011), Kocabas (2013), and Moloney and Horne<br>(2015)
	17. Cooperation with citizens and companies for resilience and low GHG emissions	Williams et al. (2010), Whiteman et al. (2011), Wamsler and Brink (2014), and Vedeld et al. (2015)
	18. Adaptation considerations inherent in urban planning	Wamsler et al. (2013) and Rosenzweig and Solecki (2014)
	19. Increase share of green–blue infrastructure	Lovell and Taylor (2013), Ristic´ et al. (2013), and Perales-Momparler et al. (2015)
	20. Holistic flood risk management	Ward et al. (2011)

	21. Inter-municipal cooperation and<br>learning for resilience and low GHG	Giest and Howlett (2013), Affolderbach and Schulz (2016), and Hoppe et al. (2016)
	22. Adaptation of tourism in a changing<br>climate	Luthe and Wyss (2016)
Agriculture and forestry	23. Decrease GHG emissions from agriculture and forestry	Knuth (2010) and Lwasa et al. (2014)
	24. Enhance usage of locally produced food<br>and timber	Knuth (2010) and Lwasa et al. (2014)
	25. Adaptation of agriculture and forestry on own land or info. to producers	Roberts and O'Donoghue (2013)<br>and Ray et al. (2014)
	26. Facilitate urban and peri-urban agriculture<br>and gardening	Lwasa et al. (2014)
Biodiversity	27. Increase the share of organic food (schools, health care)	Stehfest et al. (2009) and Frenette et al. (2017)
	28. Mainstream ecosystem-based adaptation in environmental management	Wamsler et al. (2014), McPhearson et al. (2015), and Wamsler et al. (2016)
	29. Preserve biological diversity in a changing<br>climate	Cloern et al. (2011)
Health	30. Identify vulnerable groups (for heat,<br>flooding, etc.)	Martinez et al. (2011), Adetokunbo and Emeka (2015), and Carter et al. (2015)
	31. Adaptation to avoid health related impacts (for heat, flooding, etc.)	Martinez et al. (2011), Stone et al. (2013), and Boezeman and Kooij (2015)
	32. Adapt management practices in health and social care	Martinez et al. (2011)
Water infrastructure	33. Assess vulnerability of and adapt urban<br>storm and waste water systems	Van Dijk et al. (2014), Koop and van Leeuwen (2015), and Perales-Momparler et al. (2015)
	34. Assess vulnerability of and adapt drinking<br>water systems	Carden and Armitage (2013), Sara and Baud (2014), and Koop and van Leeuwen (2015)
	35. Secure reserve water (in case of, e.g.,<br>drought or contamination)	Sara and Baud (2014) and Kumar et al. (2015)
	36. Decrease leakage in water infrastructure	Koop and van Leeuwen (2015) and Neumann et al. (2015)

Complete list of references to the identified key urban climate transition activities presented in Table S5

Adetokunbo, I., and M. Emeka. 2015. Urbanization, housing, homelessness and climate change adaptation in Lagos, Nigeria: Lessons from Asia. *Journal of Design and Built Environment* 15: 15–28.

Affolderbach, J., and C. Schulz. 2016. Mobile transitions: Exploring synergies for urban sustainability research. *Urban Studies* 53: 1942–1957.

Boezeman, D., and H.J. Kooij. 2015. Heated debates: The transformation of urban warming into an object of governance in the Netherlands. In *Evolutionary Governance Theory: Theory and Applications*, ed. R. Beunen, K. Van Assche, and M. Duineveld, 185–203. Switzerland: Springer International Publishing.

Burch, S., Y. Herbert, and J. Robinson. 2015. Meeting the climate change challenge: A scan of greenhouse gas emissions in BC communities. *Local Environment* 20: 1290–1308.

Busch, H., and K. McCormick. 2014. Local power: Exploring the motivations of mayors and key success factors for local municipalities to go 100% renewable energy. *Energy, Sustainability and Society* 4: 1–15.

Carden, K., and N.P. Armitage. 2013. Assessing urban water sustainability in South Africa - Not just performance measurement. *Water SA* 39: 345–350.

Carter, J.G., G. Cavan, A. Connelly, S. Guy, J. Handley, and A. Kazmierczak. 2015. Climate change and the city: Building capacity for urban adaptation. *Progress in Planning* 95: 1–66.

Cloern, J.E., N. Knowles, L.R. Brown, D. Cayan, M.D. Dettinger, T.L. Morgan, D.H. Schoellhamer, M.T. Stacey, M. van der Wegen, R.W. Wagner, and A.D. Jassby. 2011. Projected evolution of California's San Francisco bay-delta-river system in a century of climate change. *PLoS ONE* 6(9): e24465. http://dx.doi.org/10.1371/journal.pone.0024465

Colombier, M., and J. Li. 2012. Shaping climate policy in the housing sector in northern Chinese cities. *Climate Policy* 12: 453–473.

Corvellec, H., M.J.Z. Campos, and P. Zapata. 2013. Infrastructures, lock-in and sustainable urban development: The case of waste incineration in the Gothenburg Metropolitan Area. *Journal of Cleaner Production* 50: 32–39.

Davoudi, S., P. Zhao, and E. Brooks. 2014. Retrofitting cities for low-carbon urban futures in Europe and China. *disP - The Planning Review* 50: 6–10.

van Dijk, E., J. Van Der Meulen, J. Kluck, and J.H.M. Straatman. 2014. Comparing modelling techniques for analysing urban pluvial flooding. *Water Science and Technology* 69: 305–311.

Eames, M., T. Dixon, T. May, and M. Hunt. 2013 City futures: Exploring urban retrofit and sustainable transitions. *Building Research and Information* 41: 504–516.

Frenette, E., O. Bahn, and K. Vaillancourt. 2017. Meat, dairy and climate change: Assessing the long-term mitigation potential of alternative agri-food consumption patterns in Canada. *Environmental Modeling and Assessment* 22: 1–16.

Giest, S., and M. Howlett. 2013. Comparative climate change governance: Lessons from European transnational municipal network management efforts. *Environmental Policy and Governance* 23: 341–353.

Glaas, E., T.-S. Neset, E. Kjellström, and A.-J. Almås. 2015. Increasing house owners adaptive capacity: Compliance between climate change risks and adaptation guidelines in Scandinavia. *Urban Climate* 14: 41–51.

La Greca, P., L. Barbarossa, M. Ignaccolo, G. Inturri, and F. Martinico. 2011. The density dilemma. A proposal for introducing smart growth principles in a sprawling settlement within Catania Metropolitan Area. *Cities* 28: 527–535.

Hawkey, D., J. Webb, and M. Winskel. 2013. Organisation and governance of urban energy systems: District heating and cooling in the UK. *Journal of Cleaner Production* 50: 22–31.

Heiskanen, E., R. Lovio, and M. Jalas. 2011. Path creation for sustainable consumption: promoting alternative heating systems in Finland. *Journal of Cleaner Production* 19: 1892–1900.

Hofmann, M., N.D. Müller, C.J. Stankiewicz, A. Pfnür, and H.J. Linke. 2015. The effects of knowledge orders on climate change policy in urban land management and real estate management: A case study of three German cities. *Urban Research and Practice* 8: 336–353.

Hoppe, T., A. van der Vegt, and P. Stegmaier. 2016. Presenting a framework to analyze local climate policy and action in small and medium-sized cities. *Sustainability* 8: 847; doi:10.3390/su8090847

Johansson, T., M. Vesterlund, T. Olofsson, and J. Dahl. 2016. Energy performance certificates and 3-dimensional city models as a means to reach national targets - A case study of the city of Kiruna. *Energy Conversion and Management* 116: 42–57.

Knuth, S.E. 2010. Addressing place in climate change mitigation: Reducing emissions in a suburban landscape. *Applied Geography* 30: 518–531.

Kocabas, A. 2013. The transition to low carbon urbanization in Turkey: Emerging policies and initial action. *Habitat International* 37: 80–87.

Koop, S.H.A., and C.J. van Leeuwen. 2015. Application of the improved city blueprint framework in 45 municipalities and regions. *Water Resource Management* 29: 4629–4647.

Kumar, V., L. Del Vasto-Terrientes, A. Valls, and M. Schuhmacher. 2016. Adaptation strategies for water supply management in a drought prone Mediterranean river basin: Application of outranking method. *Science of the Total Environment* 540: 344–457.

Liu, W., C. Wang, X. Xie, A.P.J. Mol, and J. Chen. 2012. Transition to a low-carbon city: Lessons learned from Suzhou in China. *Frontiers of Environmental Science and Engineering in China* 6: 373–386.

Lovell, S.T., and J.R. Taylor. 2013. Supplying urban ecosystem services through multifunctional green infrastructure in the United States. *Landscape Ecology* 28: 1447–1463.

Luthe, T., and R. Wyss. 2016. Resilience to climate change in a cross-scale tourism governance context: A combined quantitative-qualitative network analysis. *Ecology and Society* 21: 27. http://dx.doi.org/10.5751/ES-08234-210127

Lwasa, S., F. Mugagga, B. Wahab, D. Simon, J. Connors, and C. Griffith. 2014. Urban and peri-urban agriculture and forestry: Transcending poverty alleviation to climate change mitigation and adaptation. *Urban Climate* 7: 92–106.

Martinez, G.S., C. Imai, and K. Masumo. 2011. Local heat stroke prevention plans in Japan: Characteristics and elements for public health adaptation to climate change. *International Journal of Environmental Research and Public Health* 8: 4563–4581.

McPhearson, T., E. Andersson, T. Elmqvist, N. Frantzeskaki. 2015. Resilience of and through urban ecosystem services. *Ecosystem Services* 12: 152–156.

Mejía-Dugand, S., O. Hjelm, L. Baas, and R. Ríos 2013. Lessons from the spread of bus rapid transit in Latin America. *Journal of Cleaner Production* 50: 82–90.

Sara, M.L., and I. Baud. 2014. Knowledge-building in adaptation management: Concertación processes in transforming Lima water and climate change governance. *Environment and Urbanization* 26: 505–524.

Moloney, S., and R. Horne. 2015. Low carbon urban transitioning: From local experimentation to urban transformation? *Sustainability* 7: 2437–2453. https://doi.org/10.3390/su7032437

Neumann, M. 2005. The compact city fallacy. *Journal of Planning Education and Research* 25: 11–26.

Neumann, M.B., J. Rieckermann, T. Hug, and W. Gujer. 2015. Adaptation in hindsight: Dynamics and drivers shaping urban wastewater systems. *Journal of Environmental Management* 151: 404–415.

Oshiro, K., and T. Masui. 2015. Diffusion of low emission vehicles and their impact on CO2 emission reduction in Japan. *Energy Policy* 81: 215–225.

Perales-Momparler, S., I. Andrés-Doménech, J. Andreu, and I. Escuder-Bueno. 2014. A regenerative urban storm water management methodology: The journey of a Mediterranean city. *Journal of Cleaner Production* 109: 174–189.

Ray, D., S. Bathgate, D. Moseley, P. Taylor, B. Nicoll, S. Pizzirani, and B. Gardiner. 2014. Comparing the provision of ecosystem services in plantation forests under alternative climate change adaptation management options in Wales. *Regional Environmental Change* 15: 1501–1513.

Ristić, R., B. Radić, G. Trivan, and I. Malusevic. 2013. “Blue-green” corridors as a tool for erosion and stream control in highly urbanized areas - Case study of Belgrade city. *Spatium* 30: 18–22.

Roberts, D., and S. O’Donoghue. 2013. Urban environmental challenges and climate change action in Durban, South Africa. *Environment and Urbanization* 25: 299–319.

Rosenzweig, C., and W. Solecki. 2014. Hurricane Sandy and adaptation pathways in New York: Lessons from a first-responder city. *Global Environmental Change* 28: 395–408.

Shukla, P.R., and S. Dhar. 2015. Energy policies for low carbon sustainable transport in Asia. *Energy Policy* 81: 170–175.

Stehfest, E., L. Bouwman, D.P. van Vuuren, M.G.J. den Elzen, B. Eickhout, and P. Kabat. 2009. Climate benefits of changing diet. *Climatic Change* 95: 83–102.

Stone, B., J. Vargo, P. Liu, Y. Hu, and A. Russell. 2013. Climate change adaptation through urban heat management in Atlanta, Georgia. *Environmental Science and Technology* 47: 7780–7786.

Uyarra, E., and S. Gee. 2013. Transforming urban waste into sustainable material and energy: The case of Greater Manchester. *Journal of Cleaner Production* 50: 101–110.

Vedeld, T., A. Coly, N.M. Ndour, and S. Hellevik. 2015. Climate adaptation at what scale? Multi-level governance, resilience, and coproduction in Saint Louis, Senegal. *Natural Hazards* 82: 173–199.

Wamsler, C., E. Brink, and C. Rivera. 2013. Planning for climate change in urban areas: From theory to practice. *Journal of Cleaner Production* 50: 68–81.

Wamsler, C., and E. Brink. 2014. Planning for climatic extremes and variability: A review of Swedish municipalities’ adaptation responses. *Sustainability* 6: 1359–1385.

Wamsler, C., C. Luederitz, and E. Brink. 2014. Local levers for change: Mainstreaming ecosystem-based adaptation into municipal planning to foster sustainability transitions. *Global Environmental Change* 29: 189–201.

Wamsler, C., and E. Brink. 2015. The role of individual adaptive practices for sustainable adaptation. *International Journal of Disaster Resilience in the Built Environment* 6: 6–29.
Wamsler, C., L. Niven, T. H. Beery, T. Bramryd, N. Ekelund, K. I. Jönsson, A. Osmani, T. Palo, and S. Stålhammar. 2016. Operationalizing ecosystem-based adaptation: Harnessing ecosystem services to buffer communities against climate change. *Ecology and Society* 21: 31. http://dx.doi.org/10.5751/ES-08266-210131
Ward, P.J., W.P. Pauw, M.W. van Buuren, and M.A. Marfai. 2013. Governance of flood risk management in a time of climate change: The cases of Jakarta and Rotterdam. *Environmental Politics* 22: 518–536.
Whiteman, G., D.R. de Vos, F.S. Chapin, V. Yli-Pelkonen, J. Niemelä, and B.C. Forbes. 2011. Business strategies and the transition to low-carbon cities. *Business Strategy and the Environment* 20: 251–265.
Williams, K., J.L.R Joynt, and D. Hopkins. 2010. Adapting to climate change in the compact city: The suburban challenge. *Built Environment* 36: 105–115.
Zaman, A., and S. Lehmann. 2013. The zero waste index: A performance measurement tool for waste management systems in a zero waste city. *Journal of Cleaner Production* 50: 123–132.
